# Management and Reconstruction in the Breast Cancer Patient With a Fungating T4b Tumor

**Published:** 2015-09-09

**Authors:** Aditya Sood, Lily N. Daniali, Kameron S. Rezzadeh, Edward S. Lee, Jonathan Keith

**Affiliations:** Department of Surgery, Division of Plastic Surgery, Rutgers University–New Jersey Medical School, Newark, NJ

**Keywords:** breast cancer, reconstructive technique, advanced breast cancer, breast reconstruction, ulcerative breast cancer

## Abstract

**Background:** A subset of women with locally advanced breast cancer presented with fungating tumor mass eroding and infiltrating the surrounding breast skin (T4b breast cancers). These patients often have chronic pain, large open wounds, frequent infections, malodorous drainage, social isolation, and general debilitation that present enormous therapeutic challenges. Because of the advanced nature of the disease, palliation, while minimizing recovery time and maximizing quality of life, is essential. **Methods:** From 2009 to 2014, a total of 12 consecutive patients underwent resection of fungating T4b breast tumors and subsequent chest wall reconstruction. Demographic, socioeconomic, and clinical data were collected retrospectively. **Results:** Fifty percent of women had distant metastases at the time of reconstruction, and 17% of women presented to the emergency department in a hemodynamically unstable condition in either hemorrhagic shock or septic shock, necessitating delay of reconstruction for up to 1 week. Mean wound size for reconstruction was 473 cm^2^. Reconstructive procedures included split-thickness skin grafting and thoracoepigastric advancement, latissimus dorsi, trapezius, and extended transverse and vertical rectus abdominis flaps. Postoperative survival ranged from 98 to 172 days (mean = 127 days), with 9 patients currently living. Seventy-five percent of patients had improved pain and reduced wound care needs after reconstruction. Postoperative reconstruction-specific complications occurred in 33% of cases, with 1 patient requiring a second operating room visit. **Conclusions:** Women with fungating T4b breast cancer tumors often present with metastatic disease and have significant need for pain and wound palliation. The reconstructive techniques performed are reliable, efficacious in palliating pain, and reducing wound care needs and have low complication rates.

Locally advanced breast cancer (LABC) is the presenting form of breast cancer in 5% to 10% of cases in economically developed countries and 20% to 25% of cases worldwide.^1^^-^3 The term “LABC” encompasses a heterogeneous group of diseases including all T3-T4 cancers. A subpopulation of patients with LABC is patients with local growth of the tumor resulting in erosion and infiltration of breast skin (T4b breast cancers). T4b tumors may grow to become fungating masses, extending above the skin surface with a fungus or cauliflower-like appearance.^4^ Chronic pain, frequent infection, foul smell, social isolation, and general debilitation are persistent therapeutic challenges contributing to a profound impact on quality of life and femininity in this patient population.^5^^,^^6^ For this subset of patients, a definitive cure is unlikely and palliation is difficult.

The optimal surgical management of LABC continues to be a source of controversy.^1^^,^^7^^-^9 In patients with skin involvement that has progressed to frank T4b disease, neoadjuvant chemotherapy is frequently utilized to allow for the conversion of mastectomy to breast-conserving surgery.^7^ However, there is only limited data regarding breast-conserving therapy in T4b breast cancer. Of note, a higher T-status according to the TNM system is not an absolute contraindication for breast-conserving therapy as long as surgical margins are properly maintained and postoperative radiation is utilized.^10^

Studies on breast reconstruction following the extirpation of LABC are limited but are generally in favor of immediate reconstruction regardless of cancer stage.^9^ The objective of breast reconstruction, irrespective of the mastectomy procedure preceding it, is to improve the remaining quality of life for the patient.^11^ In the past, good locoregional control has been achieved using immediate autologous tissue breast construction in stage III/IV breast cancer.^12^ Immediate breast reconstruction following neoadjuvant chemotherapy in patients with LABC does not result in increased complication rates, nor does it delay the start of adjuvant treatment, and can therefore be performed safely within this patient population; in addition, the effects of postmastectomy radiation on the reconstructed breast are now known to be less significant than previously reported.^9^^,^^13^^,^^14^

The surgical and reconstructive management of fungating T4b tumors has never been examined. We use experience gained through a series of cases treated at our institution to develop recommendations for the optimal treatment of patients presenting with these debilitating lesions. The purpose of this study was to offer the first specific algorithm for the assessment and reconstructive management of the fungating T4b breast.

## MATERIALS AND METHODS

### Study design and population

This work has been approved by the institutional review board at Rutgers University–New Jersey Medical School. We performed a retrospective review of the electronic medical records of patients treated from 2011 to 2014 at the University Hospital in Newark, NJ. A total of 12 patients underwent reconstructive surgery following resection of fungating T4b breast tumors.

### Demographics and preoperative clinical data

Data were collected from patient electronic medical records. Clinical characteristics of interest included age, chief complaint, location of first presentation, and reasons for delayed presentation. Tumor characteristics including tumor dimensions and nature of wound/odor were also analyzed when available. Disease features including pathologic stage and presence of distant metastases were relevant to this study. Preoperative laboratory values of interest included albumin, hemoglobin, white blood cell count, platelet count, international normalized ratio, and creatinine.

### Postoperative clinical data

Administration of adjuvant chemotherapy or postoperative radiation was documented. Types of oncologic and plastic surgery performed were recorded and analyzed. Postoperative follow-up, disposition, and survival were also noted.

## RESULTS

### Patient data

Twelve patients underwent a total of 14 breast tumor resections and 13 pedicled flap reconstructions with or without split-thickness skin grafting of complex chest wall defects. Patient demographic data are listed in Table 1. The mean age within our patient population was 52.6 years, with an age range of 33 to 74 years. Seven of the 12 patients in this study were African American; 4 patients reported Hispanic ethnicity. Five of the 12 patients were English-speaking. One patient reported recent homelessness, and 5 patients were unemployed at the time of presentation.

### Preoperative assessment

Data pertaining to clinical presentation and preoperative assessment are listed in Table 2. Three patients first presented to the emergency department at the University Hospital in Newark, NJ. Seven patients initially presented to our charity care clinic. Two of the 12 patients in this study had health care insurance at the time of presentation. The most common chief complaint among these patients was the presence of an open wound (100%) or pain at the lesion site (88%). Hemodynamic instability was noted in a significant percentage of patients (17%); one patient presented in septic shock, whereas another was in hypovolemic shock as a result of blood loss from her lesion. A quarter of the patients studied reported a malodorous discharge at the time of presentation. Among those patients who cited reasons for their delayed presentation, embarrassment and fear of treatment were among the responses provided. One half of patients had a diagnosis of distant metastases by imaging at the time of presentation, whereas a minority (42%) received neoadjuvant chemotherapy prior to surgical intervention. The majority of defects were unilateral, although bilateral breast involvement was noted in 22% of cases. One patient presented to us from a psychiatric institution where she was undergoing treatment of paranoid schizophrenia, whereas another carried a preexisting diagnosis of generalized anxiety disorder.

Preoperative laboratory characteristics are listed in Table 3. The mean hemoglobin level of this cohort was 9.8, although 1 patient presented to our institution with a hemoglobin level of 4.2.

### Surgical data

Characteristics of oncologic and reconstructive management of these patients are listed in Table 4. The most common oncologic surgery performed to resect T4b breast tumors in this patient population was a modified radical mastectomy (MRM; 75%). Modified radical mastectomy with removal of the pectoralis major fascia was performed in 4 patients (33%). Axillary lymph node dissections were performed on all patients receiving MRM. Fewer patients required radical mastectomy (17%) or procedures involving chest wall resection (17%). The mean size of the chest wall defect requiring reconstruction was 473 cm^2^. The majority of initial reconstructive surgical procedures occurred immediately following tumor resection (83%), although 2 patients underwent reconstruction more than 3 days after removal of their tumor. Five of the 12 patients required 2 reconstructive surgical procedures. The thoracoabdominal advancement flap and the latissimus dorsi flap were among the most frequently used reconstructive modalities. The mean hospital stay among this group was 7 days, and a significant percentage (33%) of patients were discharged to hospice.

#### Postoperative management and outcomes

Postoperative data are listed in Tables 5–7. The mean duration of follow-up in this patient population was 93 days, with a mean postoperative survival time of 157 days. Surgical outcomes were measured until 6 weeks postoperatively. Within this time window, 2 patients experienced a wound dehiscence, 1 patient complained of recurrence of wound bleeding, and 1 patient presented to the clinic with significant drainage from her surgical site. One patient within our cohort was found to have a postoperative infection. Most patients did not receive postoperative chemotherapy (68%) or radiation (92%). A majority (75%) of our patients reported postoperative pain reduction at the surgical site, with one third of patients stating that their wound care had improved since tumor resection and breast reconstruction.

## INSTITUTIONAL CASE SERIES

### Case 1

**Figure 1:**
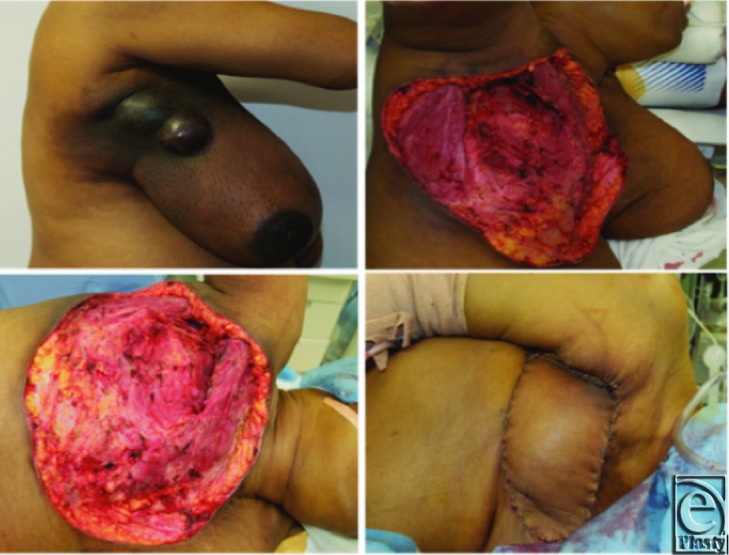
(a) Preoperative image of the right-sided fungating breast tumor. (b) Extirpative defect of the right chest wall. (c) A 884-cm^2^ defect, immediately reconstructed. (d) Immediate reconstruction with a latissimus myocutaneous flap and reverse abdominoplasty (rectus abdominis fasciocutaneous flap).

A 61-year-old woman of Haitian decent presented to the charity care clinic at the University Hospital in Newark, NJ, complaining of right breast, arm, and back pain. On physical examination, the patient was found to have a 15 × 13.5 × 11-cm right lateral breast mass with *peau d’orange* and ulceration with pruritic borders (Fig. 1*a*). She was unemployed and lived with her 2 sons, not citing a specific reason for her delayed presentation. The patient was evaluated by the medical and surgical oncology services and received a diagnosis of inflammatory breast cancer of the right breast, with right axillary lymphadenopathy and no distant metastasis, and was scheduled to receive neoadjuvant therapy prior to surgical consideration.

Approximately 6 months after receiving neoadjuvant chemotherapy, she underwent oncologic surgery of her right breast consisting of MRM and lymph node dissection (Figs. 1*b* and 1*c*). The wound measured 28.5 × 24 cm (684 cm^2^), and she immediately underwent reconstruction with a latissimus myocutaneous flap and reverse abdominoplasty (rectus abdominis fasciocutaneous flap) (Fig. 1*d*). Pathologic stage was determined to be T4bN2a with clear margins. Her hospital stay was 4 days, and she was ultimately discharged home with nursing care. She continued to receive outpatient chemotherapy.

The patient followed up in our clinic up to 8 months postoperatively. Throughout her postoperative course, she reported improved pain but claimed it was still present because of her surgery. She had no postoperative drainage, bleeding, or dehiscence. Her reconstruction was not complicated by infection, nor did she experience any complications requiring further operation from her first reconstructive surgery. Approximately 11 months after her initial surgery on her right breast, she underwent a second operation for an enlarging left breast mass considered to be a new primary site of cancer, consisting of a radical mastectomy and coverage of the defect with a pectoralis minor flap, adjacent tissue transfer of axillary skin, and placement of a negative pressure wound dressing. Her second reconstruction, on her left breast, was complicated by infection, which was treated with intravenous antibiotics. She went on to heal without any complication. Her postoperative survival time from her initial surgery was 294 days.

### Case 2

**Figure 2:**
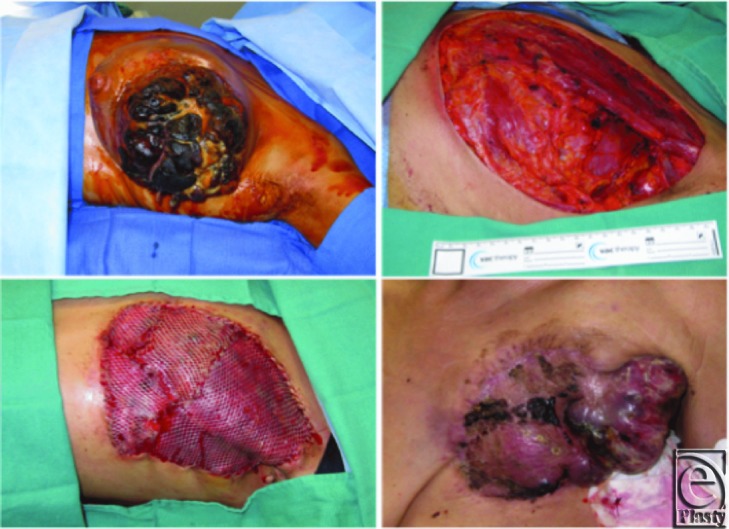
(a) Perioperative image of the left-sided fungating breast tumor. (b) A 240-cm^2^ defect, initially managed with negative pressure wound therapy. (c) Stage 2 reconstruction with split-thickness skin grafting over a 240-cm^2^ defect. (d) Two months after discharge, recurrent left breast mass protruding through graft site.

A 53-year-old woman of Hispanic descent presented to the emergency department at our institution complaining of foul-smelling discharge and pain at the left breast, as well as fatigue and a 24-lb weight loss over the prior 2 months. The patient reports that she first began to experience pain within her breast 3 months ago, at which time she began to treat herself with “alternative therapies.” On examination, she was noted to have a large, ulcerated, fungating breast mass with foul-smelling purulent drainage and bleeding (Fig. 2*a*). Her hemoglobin level on admission to the hospital was 4.2. The patient reported that she avoided seeking medical treatment of her breast mass until this admission because of fear of treatment. Her 44-year-old sister died of breast cancer after a long treatment course and multiple operations. At the time of her presentation, the patient lived at home with her mother, husband, and son.

After receiving 4 units of packed red blood cells, the patient underwent MRM with removal of the pectoralis major fascia and axillary lymph node dissection. Pathologic stage was found to be T4bN4p3a with a mass size of 19 × 15 × 12 cm. Reconstruction was performed in 2 stages, the first of which involved closure of axillary contents with the application of a negative pressure wound therapy dressing on the day of initial resection (Fig. 2*b*). The second stage of reconstruction involved a 240-cm^2^ split-thickness skin graft (Fig. 2*c*). The patient had a 15-day hospital course at our institution. She refused discharge to hospice and was instead discharged home.

The patient refused postoperative radiation therapy and missed multiple chemotherapy appointments with medical oncology. When contacted via telephone, the patient cited “excessive breast pain” as the reason for her poor follow-up. She never returned to our clinic for a postoperative examination. She subsequently revisited the emergency department 2 months after her discharge complaining of a recurrent left breast mass and discharge from the breast. Examination revealed a large fungating breast mass protruding through the graft site (Fig. 2*d*). At this point, the patient was discharged home without further surgical or medical intervention for her disease.

### Case 3

**Figure 3:**
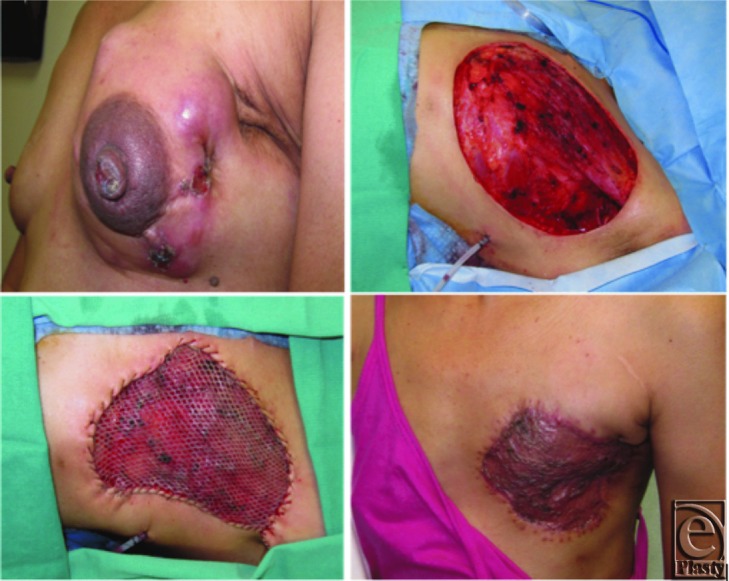
(a) Preoperative image of the left-sided fungating breast tumor. (b) A 300-cm^2^ defect, immediately reconstructed. (c) Immediate reconstruction with split-thickness skin grafting over a 300-cm^2^ defect. (d) One month postoperatively, revealing a well-healed recipient site.

A 38-year-old Hispanic woman presented to our clinic complaining of minimal pain at her chest with movement of her left arm for several years’ duration. On examination, she was found to have a large ulcerative lesion on her left lateral breast, measuring 9 × 8.4 × 4 cm and not associated with any drainage or bleeding (Fig. 3*a*). At the time of this presentation, the patient was employed as a housekeeper and lived at home with 3 children. She had noticed a left breast mass 4 years prior while in the Dominican Republic, which was finally diagnosed in the United States 2 years later after which she underwent neoadjuvant chemotherapy with an unfavorable response. The patient was evaluated by medical, surgical, and radiation oncology and received a diagnosis of invasive ductal carcinoma with metastasis. She underwent MRM and axillary lymph node dissection shortly thereafter with immediate reconstruction of the 15 × 20-cm (300 cm^2^) defect with advancement of local axillary skin and split-thickness skin grafting to the defect (Figs. 3*b*–3*d*). Pathology revealed diseased margins and a stage T4bN0 tumor.

Her postoperative course demonstrated no complications, and she was discharged home on postoperative day 6. She was followed up in our clinic regularly and stated she no longer had any pain at her surgical site as early as 2 weeks postoperatively. She received postoperative radiation to her breast subsequently due to remaining disease. She is currently living and during her last visit expressed that she would undergo the procedure again due to alleviation of her pain symptoms and no complications from the procedure.

## DISCUSSION

Despite increasing rates of screening mammography and neoadjuvant therapy within the general population, LABC continues to be the presenting form of disease in a significant percentage of patients with breast cancer.^1^^-^3^,^^15^^,^^16^ Twelve women presented to our institution with fungating T4b breast tumors over the course of a 4-year period. Patients presenting with this uncommon subtype of LABC frequently suffer from pain, malodor, social isolation, and profound effects on femininity; symptoms are usually endured for several months prior to presentation.^5^^,^^6^^,^^17^ Delayed presentation has been hypothesized to be the result of a history of a negative health care experience, health care avoidance as a coping mechanism for anxiety or pain, or barriers to access including lack of health care insurance.^5^^,^^18^ Although data on the management of LABC remain scarce, palliative reconstructive surgery has been shown to improve quality of life in patients with LABC without compromising oncologic safety or increasing complication rates.^19^^,^^20^

Women presenting with fungating T4b lesions pose unique clinical challenges to oncologic and plastic surgeons. In our study, we sought to specifically examine the extirpative and reconstructive management of fungating T4b tumors. We have demonstrated that breast reconstruction in women with these disfiguring lesions is reliable in terms of providing substantial palliation of pain and improved wound hygiene with acceptable complication rates.

Given recent studies highlighting the important psychological benefits of immediate reconstruction, as well as our desire to expedite the administration of adjuvant therapy in these patients, we sought to perform immediate reconstruction whenever possible.^21^^,^^22^ In 2 instances, patients presented to our institution in shock due to advanced medical neglect. One patient presented in septic shock and another in hypovolemic shock secondary to blood loss from her fungating breast mass. We chose to delay reconstruction in these patients until the cause of their hemodynamic instability had been addressed and achieved good outcomes in terms of patient morbidity and mortality in doing so. We believe that all patients presenting with fungating T4b lesions should be evaluated using a thorough physical examination and laboratory workup to assess for signs of hemodynamic instability prior to reconstructive management. Reconstruction should be delayed until the patient is stabilized appropriately.

Our primary objectives in the reconstruction of these patients were to cover and protect exposed vital structures, facilitate expedient wound closure, and prevent the delay of adjuvant therapy leading to optimal oncologic safety. We were also concerned with 2 principles of palliation when caring for this patient population: pain reduction and improvement of wound hygiene. At 6 weeks’ follow-up, 66% of surgical sites had healed. Less than 25% of patients complained of persistent wound drainage, and only one patient experienced wound dehiscence during her postoperative course. A majority of patients reported that wound qualities including drainage and odor had improved. Pain was reduced in 77% of patients, suggesting that the primary goals of palliation in this population were met by the reconstructive modalities utilized in this study. Despite the fact that the majority of cases examined in this series involved patients with metastatic disease, mean postoperative survival time was 157 days, with 4 patients currently living.

One goal of this study was to identify the optimal reconstructive technique for patients with fungating T4b breast tumors. Choice of reconstructive technique was based on the size and contour of the chest wall defect as well as anticipated donor site morbidity. The most commonly utilized fasciocutaneous flap in this series was the thoracoepigastric flap. This flap is based on the anterior midline overlying the upper portion of the rectus abdominis muscle and extending to the ipsilateral posterior axillary line.^23^ The thoracoepigastric flap is supplied by perforating vessels from the superior epigastric artery. This flap offers a width of 7 to 8 cm and a length of 15 to 25 cm. The authors preferred this flap for its straightforward flap harvest and reliability in the closure of small- to medium-sized chest wall defects. In several cases, a split-thickness skin graft was utilized in conjunction with the thoracoepigastric flap to cover a residual deficit. For chest wall defects between 500 and 700 cm^2^, the authors frequently opted to utilize the latissimus dorsi myocutaneous flap. The latissimus flap receives its major vascular pedicle from the thoracodorsal artery, with segmental contributions from the lumbar and intercostal arteries. Harvest of this flap is generally well tolerated, and patients infrequently report donor site morbidity. Disadvantages associated with the latissimus flap include an increased risk of donor site seroma formation.

In our study, we identified several possible psychosocial etiologies for delayed presentation among patients with fungating T4b breast tumors. Our findings suggest that patients with fungating LABC are commonly non–English-speaking, which suggests that language barriers may contribute to substantial delays in the management of breast cancer. Unemployment, homelessness, and lack of health care insurance were among the characteristics of this patient population, which support the hypothesis that psychosocial factors and, specifically, social isolation play a significant role in delay of presentation. Interestingly, our study demonstrates that fungating T4b tumors are not a disease entity associated with old age; patients who presented to us with these lesions were between 33 and 65 years of age, with an average age of 52.8 years.

Our data suggest that psychiatric illness is more prevalent among women with fungating T4b breast tumors than in the general population and may therefore be a risk factor for LABC. Our findings are in accord with a recent review by Irwin et al,^24^ which highlights the fact that 50% of patients with schizophrenia and cancer had significant delays between cancer diagnosis and initiation of treatment.^25^^,^^26^ Recent studies have also shown that once patients with schizophrenia receive a diagnosis of cancer, they are more likely to die of that cancer. Future research is needed to evaluate the length of postoperative survival in patients with LABC and psychiatric illness.

## CONCLUSIONS

While we acknowledge study limitations including retrospective design and inaccuracies inherent to chart review, we conclude that using pedicled flaps with or without split-thickness skin grafts as breast reconstruction techniques is reliable and efficacious in palliating pain and reducing wound care needs in patients with fungating T4b breast tumors. These procedures are straightforward and shortened procedures, allowing for improved quality of life in these patients with enormous defects. These procedures are especially important in such a patient population that presents with poor nutritional status and a history of hemodynamic instability. Patients presenting in shock due to advanced medical neglect should be stabilized prior to reconstructive management. Psychiatric illness and non–English-speaking status are among the risk factors for delayed presentation in this patient population.

## Figures and Tables

**Table 1:** T4b fungating breast tumor patient demographic and social characteristics

Variable	
No. of patients, *n*	12
Age, y	
Mean	52.6
Range	33-74
Race, *n* (%)	
African American	7 (50)
Caucasian	1 (8)
Hispanic	4 (33)
Social history, *n* (%)	
Recent US immigrant (<2 y)	1 (8)
Recent homelessness (<1 y)	1 (8)
Currently unemployed	5 (42)
English speaking	5 (42)
Living alone	4 (33)

**Table 2:** Clinical presentation and preoperative assessment of the patient with a fungating T4b breast tumor

Variable	*n* (%)
Location of first presentation	
Emergency department	3 (25)
Charity care clinic/community clinic	7 (58)
Private office	2 (17)
Chief complaint on presentation	
Open wound and/or skin involvement	12 (100)
Pain	8 (66)
Malodorous drainage	3 (25)
Breast mass increasing in size	3 (25)
Patients reason for delayed presentation	
Embarrassment	2 (17)
Psychiatric condition	3 (25)
Prior misdiagnosis	1 (8)
Fear of treatment	3 (25)
Tumor size, cm2	
Mean	810.2
<300	6 (50)
300-600	2 (17)
>600	3 (25)
Hemodynamic instability on arrival	2 (17)
Neoadjuvant chemotherapy	4 (42)
Neoadjuvant radiotherapy	2 (17)
Extent of disease as assessed by imaging	
Locoregional spread	3 (25)
Distant metastasis	6 (50)

**Table 3:** Preoperative laboratory characteristics

Variable	Mean	SD
Albumin	3.4	1.0
Hemoglobin	9.8	2.6
WBC	12.8	10.5
Platelets	316.9	139.8
INR	1.1	0.17
CRP	0.65	0.15

WBC indicates white blood cell; INR, international normalized ratio; and CRP, C-reactive protein.

**Table 4:** Characteristics of oncologic and reconstructive management of T4b fungating breast tumors

Variable	*n* (%)
*Oncologic management*	
Breast	
Modified radical mastectomy with axillary lymph node dissection	9 (75)
Radical mastectomy	2 (17)
Chest Wall	
Anterior pectoralis major fascia resected	4 (33)
Additional chest wall muscles resected	2 (17)
*Pathology*	
Breast specimen with clear margins (R0)	7 (58)
*Reconstructive surgery*	
Area requiring reconstruction, cm2	
Mean	473
>300	2 (17)
300-600	6 (50)
601-900	4 (33)
Procedures performed	
Thoracoepigastric advancement flap	4 (33)
Latissimus dorsi flap	5 (41)
Trapezius flap	1 (8)
Extended vertical and transverse rectus abdominis flap	1 (8)
Split-thickness skin graft	6 (50)
Timing of reconstructive surgery	
Immediate	10 (83)
Delayed	2 (17)
Total no. of reconstructive surgical procedures	
1	7 (58)
2	5 (41)
Disposition	
Home	8 (66)
Hospice	3 (25)
Psychiatric institution	1 (8)

**Table 5:** Postoperative duration of follow-up, length of stay, and postoperative survival

	Range (Mean) or *n* (%)
Duration of follow-up, d	28-290 (93)
% presenting for outpatient follow-up	11 (92)
% no show/lost to follow-up	1 (8)
Length of hospital stay, d	1-19 (7)
Postoperative survival, d	46-172 (157)

**Table 6:** Postoperative outcomes

	*n* (%)
Surgical site at 6-wk follow-up	
Healed	7 (58)
Open wound requiring dressings	3 (25)
Not reported	2 (17)
Postoperative pain palliation	
Reduced pain	9 (75)
Persistent or increased pain	2 (17)
Not reported	1 (8)
Postoperative wound palliation	
Improved wound qualities (odor, drainage)	10 (84)
Unimproved wound qualities	1 (8)
Not reported	1 (8)
Adjuvant therapy	
Chemotherapy	4 (33)
Radiation	1 (8)

**Table 7:** Postoperative complications

	*n* (%)
Persistent wound drainage	2 (17)
Dehiscence requiring operating room revision	1 (8)
Infection requiring course of antibiotics	1 (8)
